# Changes in the regional homogeneity of resting-state magnetic resonance imaging in perimenopausal women

**DOI:** 10.1186/s12905-020-01171-7

**Published:** 2021-01-28

**Authors:** Min Liu, Hui Yang, Jian Qin, Qianqian Yao, Guihua Yang, Jiang Li

**Affiliations:** Department of Radiology, The Second Affiliated Hospital of Shandong First Medical University, No.366 Taishan Street, Tai’an, Shandong China

**Keywords:** Perimenopause, Regional homogeneity, Functional magnetic resonance imaging, Resting-state, Spontaneous activity

## Abstract

**Background:**

There is a noticeable lack of systematic researches on evaluating the correlation between serum estrogen levels and changes in brain functional areas of perimenopausal women.The aim of this study is to investigate the regional spontaneous brain activity changes in perimenopausal women.

**Methods:**

Based on the resting-state functional magnetic resonance imaging datasets acquired from 25 perimenopausal women and 20 healthy women of reproductive age, a two-sample t-test was performed on individual normalized regional homogeneity (ReHo) maps. Relationships between abnormal ReHo values and the self-rating anxiety scale (SAS), the self-rating depression scale (SDS) were investigated with Pearson correlation analysis. We also investigated the correlation between abnormal ReHo values and serum estrogen level.

**Results:**

In the perimenopausal group, we found increased ReHo in the right posterior cerebellum (region 2), left middle frontal gyrus and left middle cingulate gyrus ($P<0.05$). Additionally, the ReHo values in left middle frontal gyrus and leftt middle cingulate gyrus showed positively significant correlation with the SAS, SDS scores. On the contrary, there was no significant correlation between the ReHo value in right posterior cerebellum and SDS, SAS scores. In the perimenopausal group, the ReHo values in the left middle frontal gyrus and left middle cingulate gyrus were negatively correlated with the serum estrogen level ($P<0.05$).

**Conclusion:**

The results of this preliminary study have suggested that abnormal spontaneous activities of multiple brain regions during resting state was already altered in perimenopausal women. Alterative activities might be related to emotional regulation deficits and cognitive impairment, and might potentially represent the neural mechanism underlying perimenopausal period.

## Background

Perimenopause is defined as the transition of women from normal menstruation to amenorrhea, which marked by irregular menstruous cycles and fluctuations in ovarian hormones. It is a transitional state of nervous system, including disorders of the estrogen regulatory system, such as temperature regulation, circadian rhythm changes, vasomotor symptoms and sleep disturbances, which are recognized to be factors of impairing cognitive function and mood [1]. The risk of mood disorders with a key stage of psychological disorders such as depression and anxiety has been increased in perimenopausal women, and it is a critical period for women to develop Alzheimer’s disease [2]. Estrogen, regarded as the “master regulator” of a woman’s brain and body’s metabolic system, regulate glucose transport, aerobic glycolysis and mitochondrial function to generate ATP [3]. It gradually decreased with the prolongation of perimenopause. Emotional instability and cognitive impairment may be associate with such hormonal imbalances [4, 5], which can also lead to functional or structural changes in certain brain regions. However,to the best of our knowledge, there is a noticeable lack of systematic researches on evaluating the correlation between serum estrogen levels and changes in brain functional areas of perimenopausal women.

The frontal cortex and posterior cingulate cortex showed consistent biomarker abnormalities in peri- and postmenopausal women [6]. Lu et al. found that the gray matter volume in multiple brain regions of perimenopausal women decreased [7]. However, previous studies of perimenopausal women mainly focused on the changes in brain structure and morphology, not yet revealed the overall changes in the spontaneous activity of whole brain,which did not point out abnormal area of the brain accurately. In this prospective study, we hypothesized that the spontaneous neural activity of the whole brain would change in perimenopausal women. To testify this hypothesis, we (1) characterized and compared the ReHo [8] based on rs-fMRI between perimenopausal and normal reproductive age women to investigate altered spontaneous regional brain activity in perimenopausal women; (2) examined the psychological status and serum estrogen levels of perimenopausal women, with further discussing the correlation between spontaneous brain activity changes in each region and estrogen levels and psychological status, which will be helpful to yield a more comprehensive understanding of pathophysiological changes in perimenopausal women.

## Methods

### Subjects

The study was approved by the ethics review committee of the second affiliated hospital of shandong first medical university. Informed written consent was obtained from each participant. The subjects who met the inclusion criteria in this study were recruited from June 2017 to September 2019 at the surrounding community by posting recruitment notice.

The perimenopausal group inclusion criteria: (1) a diagnosis of perimenopause based on the Stages of Reproductive Aging Workshop (STRAW) + 10: consecutive menstrual cycle differences greater than 7 days or the occurrence of amenorrhea of 60 days or longer [9]; (2) female, right handed, age ranges from 45 to 54 years old; (3) have a uterus and at least one ovary; (4) have no diabetes, vascular diseases, hypertension and smoke history; (5) no fMRI scan contraindications, such as pacemakers, metal dentures or orthodontics. Exclusion criteria: (1) severe psychiatric disorders, such as dementia, episode, epilepsy, major depression; (2) significant neurological illness, such as significant head trauma, tumor,meningitis or central nervous system (CNS) inflammatory lesions and vascular complications clues; (3) surgical menopause such as history of hysterectomy, bilateral ovariectomy, genital tumors and vaginal bleeding of unknown etiology; (4) aphasia, deafness and blindness and others unable to cooperate with the examination; (5) centrally acting medications, intermittent estrogen or phytoestrogen supplements. People with one of the above conditions will be excluded from this experiment.

Finally, twenty five perimenopause women with ages range from 45 to 52 years (mean age 50.76 years, SD = 2.72 years) were enrolled as the perimenopausal group. In addition, we also recruited twenty reproductive subjects from 32 to 45 years (mean age 36.7 years, S.D = 8.29 years) with regular menstrual cycles at the surrounding community as the control group,who were matched in education and hand dominant with perimenopausal group.

### General information and psychological assessment

The subjects’ age, age at menarche, estrogen (E2) level and education level were collected. Before undergoing the MRI, the Self-rating Anxiety Scale (SAS) [10] and the Self-rating Depression Scale (SDS) [11] scores were also gotten from each subject to assess them levels of anxiety and depression. SAS and SDS each have 20 items.Each item is scored on a scale of 1–4 (a little of the time, some of the time, a good part of the time,most of the time), and the total score ranges from 20 to 80. The raw score is then converted to an index score by dividing the raw score by the maximum score (80) and either expressing this as a decimal or multiplyingby 100 to express it as a whole number with an index score range of 25 to 100. The criteria for classifying anxiety and depression are the same. The severity of anxiety or depression was further classified as: (1) 50–59, mild; (2) 60–69, moderate; and (3) 70–100, severe [12, 13].

Venipuncture was scheduled between 8:00 and 10:00 AM after a 12-h fast during the early follicular phase(cycle days 2–5, termed “in window”) of all subjects. If a blood sample could not be obtained in the d2–5 window in the 60d after the visit date(usually because of irregular menstrual cycles), blood was obtained in the subsequent 30d without respect to menstrual bleeding. Serum was analyzed for E2. The ADVIA Centaur estradiol test was performed using an automated chemiluminescence immunoassay analyzer (Siemens Healthcare Diagnostics Inc, New York, USA). Reference standard: Early follicular phase of premenopausal women, estradiol reference range: 19.5–144.2 pg/mL.

### MRI data acquisition

The MRI data were acquired using a 3.0T MR imager (Discovery MR750, GE Healthcare, Milwaukee, WI, USA). For both the perimenopausal and the control group, data were all collected at days 1–3 after onset of menses.Each subject was supine with the head snugly secured using a belt and foam pads. First, routine sequence scanning was performed to exclude brain tumors, cerebral infarction, cerebral hemorrhage and other brain abnormalities.Then the BOLD signal was sequenced with a 3D-T1 BRAVO anatomical image of the entire brain structure and an EPI gradient plane echo sequence. During rs-fMRI, subjects were asked to lie with their eyes closed, not to fall asleep,and not to think of anything in particular. The rsfMRI acquisition parameters were as follows: 3D-T1 BRAVO sequence: TE = 3.2 ms, TR = 8.2 ms, TI = 450 ms, FOV = 25.6 mm 25.6 mm, matrix = 256256, layer spacing = 0 mm, layer thickness = 1 mm, layer number = 176, NEX = 1; BOLD sequence: EPI-GRE: TR = 2000 ms, TE = 20 ms, layer thickness = 3.2 mm, layer interval = 0 mm, FOV = 22 mm 22 mm, matrix= 6464, 240 time points, 41 layers.

### Data processing and ReHo calculations

Image preprocessing was performed using the data processing assistant in the rs-fMRI (DPABI) toolbox (http://rfmri.org/dpabi v3.0_171210) [14]. For each participant, the first 10 time points were discarded because of transient signal changes before magnetization reaching a steady-state and participants adapting to the fMRI noise. In order to minimize the influence of head motion, we first eliminated the subjects with more than 1.5 mm maximum displacement in any dimension and 1.5 degrees of angular motion during the entire fMRI.We then compared the motion courses of the two groups using a two-sample t-test and confirmed no statistical significance. Finally, we setted the head-motion measures and age as covariates for group-level comparisons,no subject was eliminated in this step. Then, the corrected images were spatially normalized to the Montreal Neurological Institute (MNI) EPI template in SPM8 and resampled to 3mm3mm3mm voxels, and the linear drift was removed. Individual ReHo maps were generated by calculating the Kendall’s coeffificient of concordance (KCC) of the time series of a given voxel to its nearest 26 voxels [8]. The average ReHo values of all voxels in the significant region were extracted using the rs-fMRI data analysis tool (REST, http://www.restfmri.net) [15] in the mask generated by the standardized step. Then, the resulting data were spatially smoothed with a Gaussian kernel (full-width at half-maximum [FWHM] = 6 mm).

### Statistical analyses

SPSS Statistics version 20.0 was used for statistical analysis.Quantitative parameters are expressed as the meansstandard deviation.Data were tested for normality analysis using the Kolmogorov–Smirnov test and then with the Levene test for variance homogeneity analysis.Two-sample t-test was performed to assess the differences in age, age at menarche, level of estrogen, duration of education, SAS and SDS scores between the perimenopausal and control subjects.To explore the ReHo differences between the perimenopausal and control subjects, a two-sample t-test was performed on the individual normalized ReHo maps,corrected for permutation testing with Threshold-Free Cluster Enhancement (TFCE) [16]. Spearman correlation analysis was performed between the mean ReHo values in significantly different areas and the SAS, SDS scores and the estrogen levels. *P* values of less than 0.05 were considered as statistically significant.

## Results

### Demographic and clinical characteristics

Compared with the control group, the perimenopausal group had lower estrogen level ($P=0.00$) and higher SAS ($P=0.00$) and SDS ($P=0.00$) scores.However, there were no statistically significant differences of age at menarche ($P=0.65$) and education level ($P =0.39$), as shown in Table 1.

**Table 1:** Demographic and clinical characteristics of perimenopausal and control group

Characteristic	Perimenopausal group ($n=25$)	Control group ($n=20$)	*t* value	*P* value
Age (years)	50.76 ± 2.72	36.70 ± 8.29	− 9.40	0.00
Menarche (years)	13.54 ± 1.19	13.75 ± 2.34	0.38	0.65
Estrogen (pg/ml)	31.25 ± 13.13	108.94 ± 63.76	17.23	0.00
Education (years)	10.90 ± 3.41	11.10 ± 2.17	1.57	0.39
SAS	51.83 ± 10.85	42.72 ± 5.84	− 5.34	0.00
SDS	55.47 ± 8.92	44.12 ± 6.14	- 7.45	0.00

### Regional spontaneous activity changes

**Fig. 1:**
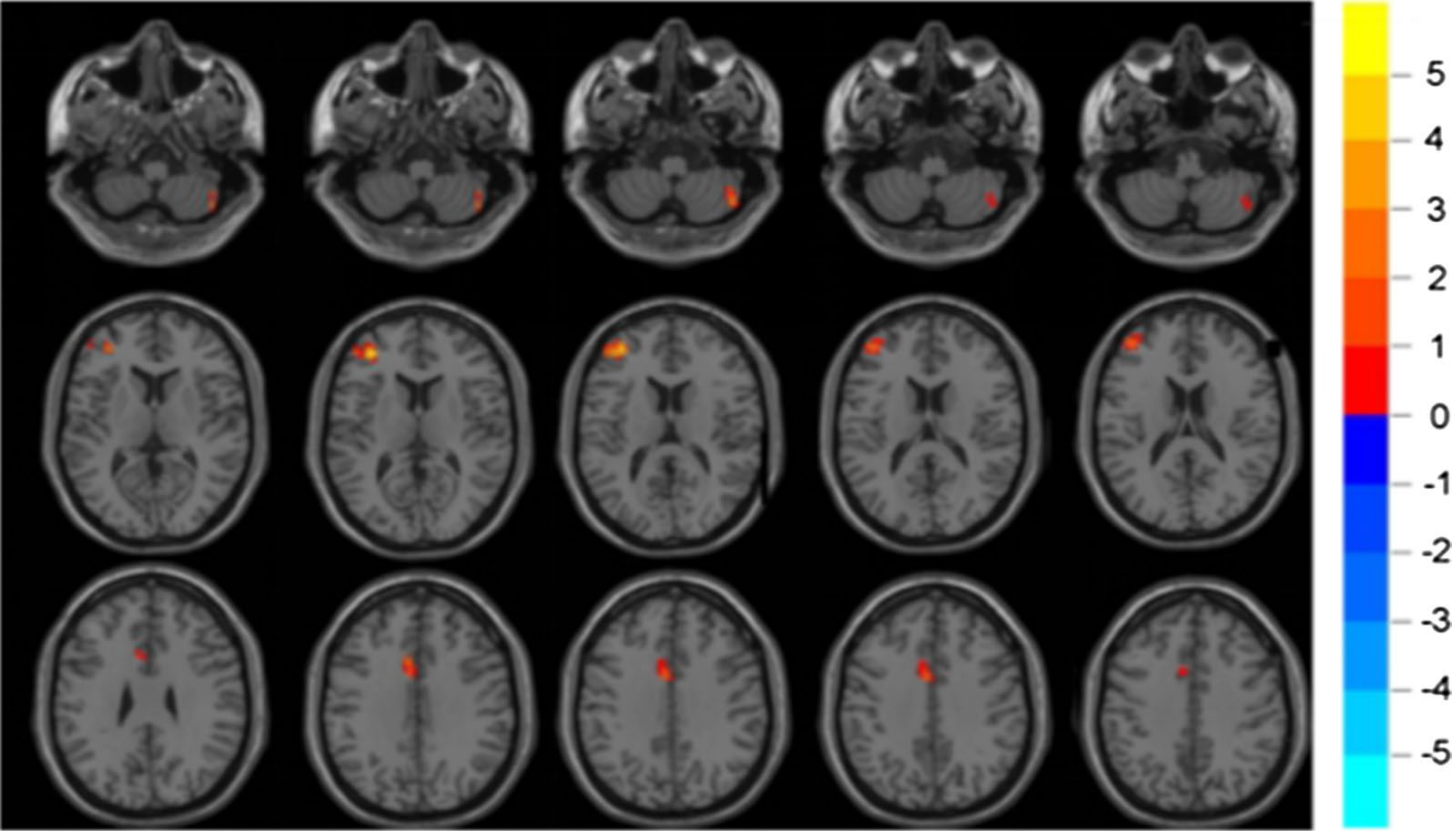
Statistically significant differences between the perimenopausal and control subjects are shown in a ReHo map of the whole-brain with MRI. The perimenopausal subjects showed a significant ReHo increase in the right posterior cerebellum (region 2), left middle frontal gyrus and left middle cingulate gyrus.A T-score bar is shown on the right. Red and green denote increases and decreases in ReHo, respectively

The rs-fMRI analysis showed that the ReHo values in the right posterior cerebellum (region 2), left middle frontal gyrus and left middle cingulate gyrus of perimenopausal women were higher than these in control subjects, with statistically significant differences ($P < 0.05$), as shown in Fig. 1 and Table 2.

**Table 2:** Brain regions with abnormal ReHo in perimenopausal group

Side	Anatomic region	MNI			Cluster size	Peak *t*-value
		*x*	*y*	*z*		
R	Cerebelum_Crus2_R	48	− 66	− 51	32	3.8982
L	Frontal_Mid_L	− 36	45	12	57	5.2187
L	Cingulum_Mid_L	− 6	15	33	39	3.9093

L: left; R: right; MNI: Montreal Neurological Institute; ReHo, regional homogeneity; Cluster$>20$, P<0.05; Permutation test and Threshold-free cluster enhancement (TFCE)

### Correlation between ReHo values in abnormal regions and SAS, SDS scores

There was no significant correlation between ReHo value in right posterior cerebellum and SDS and SAS scores. The ReHo value in left middle frontal gyrus was positively correlated with SDS score ($r=0.393, P=0.029$) and SAS score ($r=0.377, P=0.013$). The ReHo value in left middle cingulate gyrus was positively correlated with SDS score ($r=0.312, P=0.016$) and SAS score ($r=0.303, P=0.021$).

### Correlation between ReHo value in abnormal regions and estrogen level

**Fig. 2:**
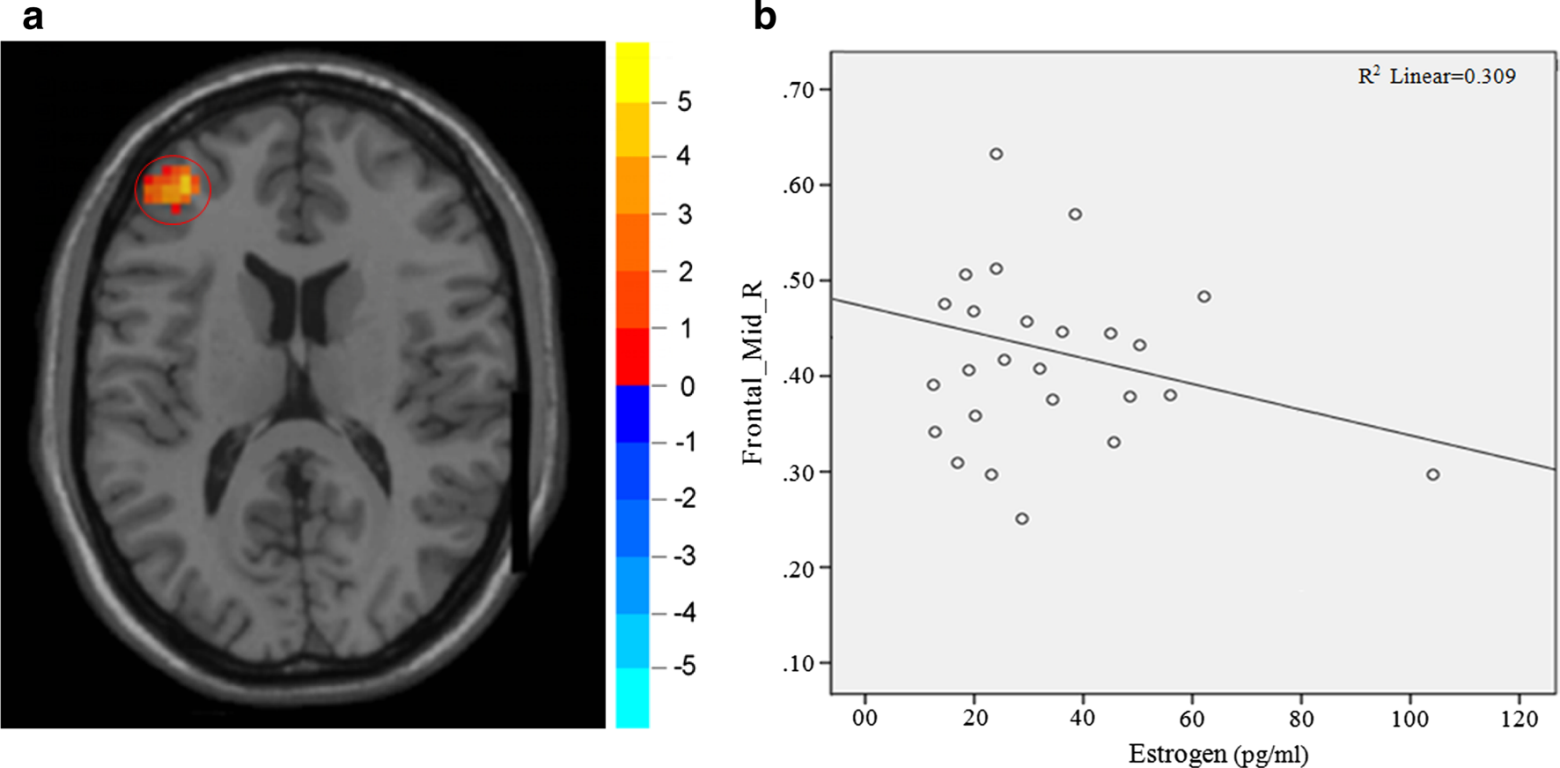
Correlation between ReHo value in left middle frontal gyrus and estrogen level (**a** on the left and **b** on the right). The ReHo value in left middle frontal gyrus was negatively correlated with estrogen level ($r=-0.556$, $P=0.025$)

**Fig. 3:**
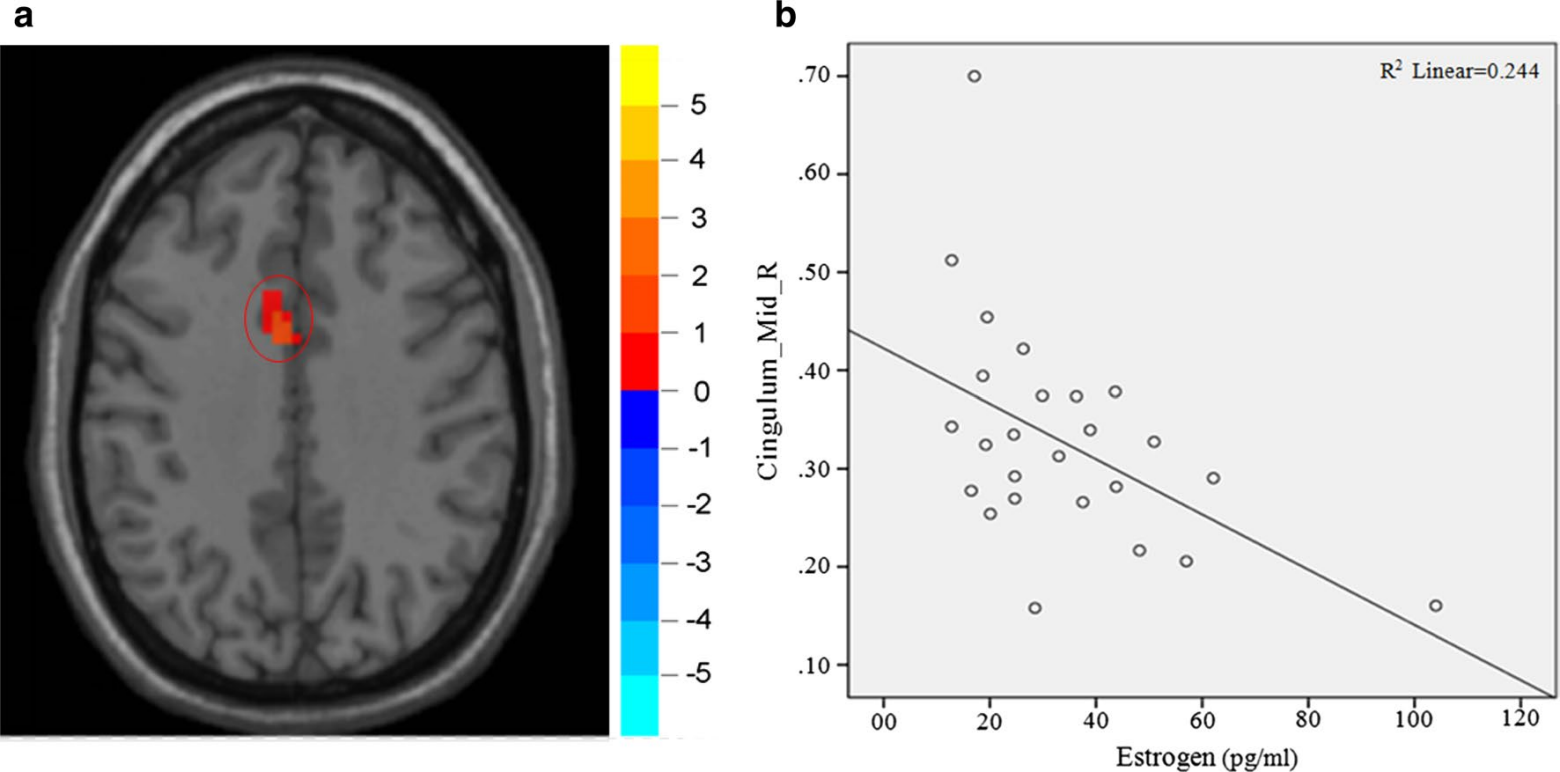
Correlation between ReHo value in left middle cingulate and estrogen level (**a** on the left and **b** on the right). The ReHo value in left middle cingulate gyrus was negatively correlated with estrogen level ($r=-0.489, P=0.038$)

In the perimenopausal group, the ReHo values in left middle frontal gyrus ($r= -0.556, P=0.025$) and left middle cingulate gyrus ($r=-0.489, P=0.038$) were negatively correlated with serum estrogen levels, as shown in Figs. 2 and 3.

## Discussion

In the present study, resting-state functional magnetic resonance imaging (rs-fMRI) ReHo analysis, basing on blood oxygen level dependency (BOLD), was applied to investigate alterations of the resting state brain activities in perimenopausal group. It was proved that the ReHo values in the right posterior cerebellum (region 2), left middle frontal gyrus and left middle cingulate gyrus of perimenopausal women were higher than these in control subjects. Additionally, the ReHo values in left middle frontal gyrus and left middle cingulate gyrus showed positively significant correlation with the SAS, SDS scores. In the perimenopausal group, the ReHo values in the left middle frontal gyrus and left middle cingulate gyrus were negatively correlated with the serum estrogen levels.

The frontal lobe is the anatomical basis of emotional activities, attention, memory, abstraction and spatial discrimination,which is involved in the formation and consolidation of memory (instantaneous memory, short-term memory and long-term memory) and is the highest nerve center of human [17, 18]. According to a review regarding attention reorienting [19], the middle frontal gyrus linked the ventral attention network (responsible for sensory-driven exogenous attention) and the dorsal attention network (responsible for goal-directed endogenous attention), thus modulated the cognitive shift between internal and external environments. Berent [20] reported that prefrontal cortex was closely related to verbal working memory, visual working memory and cognitive impairment in menopausal women. This study demonstrated that the ReHo in the left middle frontal gyrus was increased in the perimenopausal group, suggesting a decrease in the presence of cognitive function in women at the perimenopausal stage, which was consistent with weber’s finding [21]. Furthermore, Song [22]found that increased ReHo in the middle frontal gyrus (MFG) in major depressive disorder patients.Wang [23] confirmed that hyperactivation of dorsolateral prefrontal cortex in patients with generalized anxiety disorder(GAD). It suggested that the frontal lobe is related to depression and anxiety according to the above previous analysis. In this study, we found that the increase of ReHo in left middle frontal gyrus of perimenopausal women was associated with SAS, SDS scores, and further confirmed that the middle frontal gyrus was strongly associated with negative emotional activity and cognitive changes. Therefore, we speculated that the increased ReHo in left middle frontal gyrus may be associated with abnormal feelings and emotional activity, such as misgivings of the changes in menstrual cycle.

In addition, we discovered abnormity also occurred in the left middle cingulate gyrus of perimenopausal group. As an important region of the limbic system, cingulate cortex encompassed numerous and specialized subdivisions that subserved a vast array of cognitive, emotional, motor, nociceptive and visuospatial functions [24]. It also maintained strong reciprocal interconnections with lateral prefrontal cortex (BA 46/9), parietal cortex (BA 7), premotor and supplementary motor areas, and mainly served to regulate both cognitive and emotional processing [25, 26]. In the study, we had substantiated the ReHo in the left middle cingulate gyrus was increased in the perimenopausal group, suggesting that perimenopausal women have some difficulties in regulating cognitive and emotional processing. Mataix-cols et al. found that cingulate gyrus was activated in fMRI of obsessive-compulsive disorder patients after picture and sound stimulation [27]. Another study by Wang et al. also showed that the ReHo in cingulate gyrus increased in patients with primary insomnia [28]. These above studies indicated that the cingulate gyrus was associated with cognition and emotion. It was also observed in this study that abnormal ReHo value in the left middle cingulate gyrus showed a positively significant correlation with the SAS, SDS scores, that further confirmed the importance of the cingulate gyrus in cognitive and emotional processing. We boldly speculated that the ReHo in the left middle cingulate gyrus was increased due to the increasement of ReHo in the left middle frontal gyrus,because the cingulate gyrus was strong reciprocal interconnections with the lateral prefrontal cortex.

In the present study, we discovered abnormities also occurred in the right posterior cerebellum (region 2) of perimenopausal group. It is well known that cerebellum plays an important role in the execution and planning of movement [29, 30]. Nevertheless, several studies had shown that cerebellum was also involved in some of cognitive functions, such as attention and emotion processing [31, 32]. Cerebellar dysfunction, especially the posterior lobes of cerebellum, has been implicated in many rs-fMRI studies of depression [33–36], which was consistent with our study.Therefore, we hold that increased ReHo in the right posterior cerebellum (region 2) may be the important factors and early indicators of perimenopausal cognitive deficits and affective disorders.Interestingly,for perimenopausal group, increased ReHo value of cerebellum showed no significant correlation with SAS and SDS scores in the follow-up research. We speculated that the cerebellum did not participate in affecting disturbed cognitive function directly, but played a role in complex connectivity with multiple subcortical structures [37], which is called “the cerebro-cerebellar circuit”.

In addition,previous studies have suggested that a series of abnormal emotional symptoms and cognitive impairment were related to estrogen significantly in middle-aged and elderly women, and the prefrontal cortex which served episodic and working memory were riched in estrogen receptors [38]. An interventional study involving in early postmenopausal women, suggested that the effect of estrogen on serotoninergic function may be a key mechanism related to symptoms of emotion and cognition during the menopausal transition [39]. In terms of cognitive outcomes, some evidences supported the “critical window hypothesis”, which suggested that women in the earlier stages of the menopausal transition might gain cognitive benefits from hormone therapy [40]. Although there were several studies that have showed significant effects of estrogen on the prefrontal cortex (PFC), especially in terms of concentration and working memory, no study aim to explore specific action sites of perimenopausal estrogen in PFC regions, which included multiple brain regions (Brodmann’s areas 8, 9, 10, 11, 44, 45, 46, and 47,48). In the present study, we found that the increase of ReHo in left middle frontal gyrus was negatively correlated with serum estrogen levels. So it was speculated that the fluctuation of estrogen levels had the most significant effect on the frontal middle gyrus during the perimenopausal period, and the abnormal middle frontal gyrus may be an important factor leading to perimenopausal cognitive impairment. In addition, we found that the increase of ReHo in cingulate gyrus was negatively correlated with estrogen levels, considering that fluctuations in estrogen levels led to activation of 5-HT function in the cingulate cortex [41].

## Limitations

This study has some limitations. Firstly,the present study was limited by a relatively small sample size. Therefore, the results of the current study should be replicated with a larger sample. Secondly, according to Stages of Reproductive Aging Workshop (STRAW) criteria, it is divided into the early menopause transition (menstrual cycle length varying $>7$ days in consecutive cycles) and late menopause transition (occurrence of amenorrhea of 60 days or longer) as well as partial early postmenopause [9], as estrogen levels vary with the duration of perimenopause. Thirdly, at the current stage of the study, we directly compared subjects with un-matched age in perimenopausal women and healthy women of reproductive age. Further study of matched age in perimenopausal women with different levels of estrogen may help to exclude the confunding factor that possiblely from aging effect.

## Conclusion

The results of this preliminary study suggested that activity of multiple brain regions during resting state was already altered in perimenopausal women, which may be related to emotional regulation deficits and cognitive impairment,and may potentially represent the neural mechanism underlying perimenopausal period.

## Data Availability

The datasets used and/or analyzed during the current study are available from the corresponding author on reasonable request.

## Abbreviations

rs-fMRI: Resting-state functional magnetic resonance imaging
ReHo: Regional homogeneity
SAS: Self-rating anxiety scale
SDS: The self-rating depression scale
CNS: Central nervous system
BOLD: Blood oxygen level dependency
MFG: Middle frontal gyrus
GAD: Generalized anxiety disorder
PFC: Prefrontal cortex
